# Tetramethylpyrazine Inhibits Activation of Hepatic Stellate Cells through Hedgehog Signaling Pathways In Vitro

**DOI:** 10.1155/2015/603067

**Published:** 2015-08-24

**Authors:** Jue Hu, Gang Cao, Xin Wu, Hao Cai, Baochang Cai

**Affiliations:** ^1^Zhejiang Medical College, Hangzhou 310053, China; ^2^Research Center of TCM Processing Technology, Zhejiang Chinese Medical University, Hangzhou 311401, China; ^3^Department of Chinese Materia Medica, College of Pharmacy, Nanjing University of Chinese Medicine, Nanjing 210023, China

## Abstract

*Background and Aim*. Tetramethylpyrazine (TMP), a major alkaloid isolated from *Ligusticum chuanxiong*, has been reported in hepatic fibrosis models. However, the action mechanism remains unclear. In the present study, effects of tetramethylpyrazine (TMP) against hepatic stellate cell (HSC) activation as well as the possible mechanisms were evaluated. *Methods*. Western blot assay was used to detect TMP effects on protein expression of Smo, Patched, Hhip, and Gli and to investigate the effects of TMP on Cyclin D1, Cyclin E1, CDK2, Bcl-2, Bax, and caspase expression with cyclopamine supplementation. *Results*. Our results showed that TMP significantly inhibits the expression of Cyclin D1, Cyclin E1, and Cyclin-dependent kinase CDK2 and changes the HSC cycle by inhibiting the proliferation of HSC. Moreover, TMP has also been shown to decrease the expression of Bcl-2 and increase the expression of Bax in HSC-T6 cells. Furthermore, TMP can inhibit the expression of connective tissue growth factor (CTGF), and the inhibitory effect was intensified after the application of joint treatment with TMP and cyclopamine. *Conclusion*. TMP may be an effective Hh signaling pathway inhibitor for hepatic fibrosis treatment.

## 1. Introduction

Hepatic fibrosis is the compensatory reaction during the process of tissue repair secondary to various forms of chronic liver damage (related to alcohol, viral infection, or drugs) [1, 2]. In the absence of effective therapeutic measures, continued development of the disease will alter the normal liver structure and hepatic function. The liver will also develop hepatic cirrhosis because of hepatic insufficiency. Ultimately, this condition may lead to hepatocellular carcinoma. In the development process of hepatic fibrosis, activation, the proliferation of HSC provides key contributions. Therefore, the activation, proliferation, and apoptosis of HSC should be investigated to further explore the pathogenesis of hepatic fibrosis. Recent studies indicated that the hedgehog (Hh) signaling pathway may be involved in HSC activation [3, 4], which consequently affect the development of hepatic fibrosis by influencing the process that transforms HSC into myofibroblasts [5, 6].

In recent years, increasing number of studies reported about TMP in liver protection and the effects of hepatic fibrosis inhibition [7–10], which showed a promising research topic. In vivo and in vitro studies indicated that TMP can regulate the HSC cycle, thus inhibiting the proliferation of HSC or facilitating the apoptosis of HSC, as well as reducing the expression and sedimentation of extracellular matrix (ECM) [11]. TMP seemed to play the role of antifibrosis, but the action mechanism remains unclear. Although the antihepatic fibrosis effect of TMP has been investigated [12], the report did not explore whether the action mechanism was associated with the regulation of the Hh signaling pathway.

## 2. Material and Methods

### 2.1. Cell Culture and Treatment

Rat HSC-T6 cell line was purchased from the Type Culture Collection of the Chinese Academy of Sciences (Shanghai, China). The cells were cultured in DMEM medium supplemented with 10% fetal calf serum, 100 U/mL penicillin, and streptomycin. HSC-T6 cells were incubated at 37°C in a 5% CO_2_ humidified atmosphere. Culture medium was changed every 2-3 days, and HSC-T6 cells were digested with 0.25% trypsin and seeded onto a 75% confluent monolayer.

### 2.2. Cell Viability

Rat HSC-T6 cells were cultured in 96-well tissue culture plates by adding 180 *μ*L/well of a suspension of 1 × 10^4^ cells/well. After cells reached confluence, the medium was replaced with fresh medium supplemented with 10% fetal calf serum and different doses of TMP and cyclopamine. Following treatment, cell viability was determined by the use of the 3-(4,5-dimethylthiazol-2-yl)-2,5-diphenyltetrazolium bromide (MTT, Sigma) assay and the absorbance at the wavelength of 490 nm was determined using an automatic microplate reader.

### 2.3. Western Blot Analysis

The harvested rat HSC-T6 cells were lysed in ice-cold radioimmunoprecipitation (RIPA) buffer. Cell lysates were collected and centrifuged at 12,000 ×g for 15 min at 4°C. Protein concentrations were determined using a bicinchoninic acid (BCA) protein assay kit (Thermo Pierce, USA). Proteins were separated using 12% SDS-PAGE and transferred to a nitrocellulose membranes after electrophoresis. The transferred membranes were incubated with anti-CTGF, anti-*α*1(I) collagen, antifibronectin, anti-*α*-SMA, anti-Smo, anti-Patched, anti-Hhip, anti-Gli, anti-Cyclin D1, anti-Cyclin E1 (CST), anti-CDK2, anti-Bcl-2, anti-Bax, anti-PARP-1, antiprocaspase-3, antiprocaspase-9, and antiprocaspase-8. Membranes were washed and then incubated with a 1 : 5000 dilution of secondary antibody for 1 h, and the membranes were detected with the enhanced chemiluminescence system (Amersham Life Science). Proteins were visualized with ECL-chemiluminescent kit (ECL-plus, Thermo Scientific) and normalized to the signal intensity of *β*-actin.

### 2.4. Statistical Analysis

All results are expressed as the mean ± SD. Statistical significance was determined by the Student's *t*-test, and the level of *p* < 0.05 was taken as significant difference.

## 3. Results

### 3.1. TMP Inhibits Both Viability and Activation of HSC-T6 Cells

**Figure 1:**
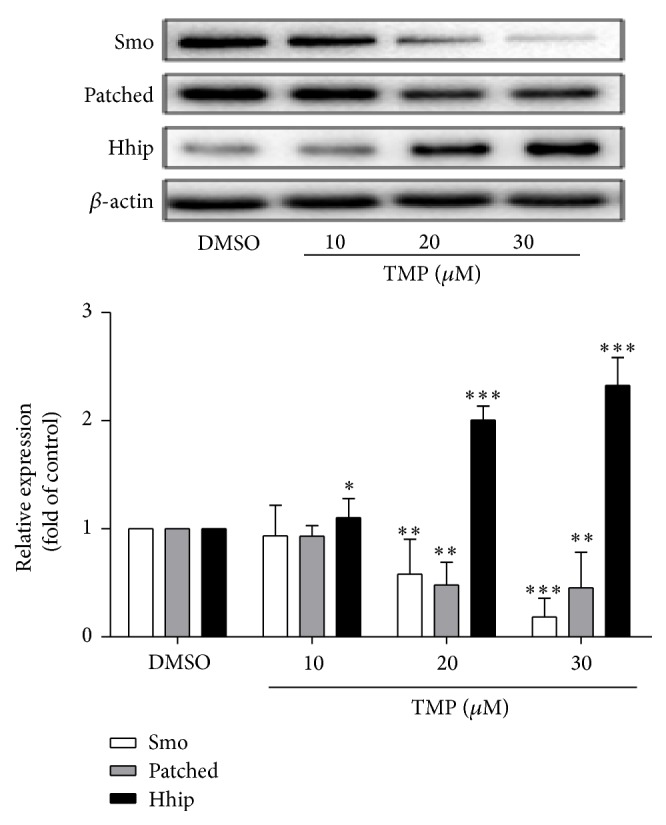
TMP inhibits both the activation of HSCs. Effects of TMP on the activities of Smo, Patched and Hhipin HSC-T6 cells. The levels of Smo, Patched, and Hhip protein levels were determined by western blot analysis and normalized by *β*-actin. Significance is defined as follows: ^*∗*^ *p* < 0.05, ^*∗∗*^ *p* < 0.01, and ^*∗∗*^ *p* < 0.001 compared with control. The DMSO was defined as control.

HSC-T6 cells were treated with different concentrations of TMP (10–80 *μ*M) and durations (0–48 h) using the MTT assay. TMP-treatment dose dependently reduces HSC-T6 cell proliferation slightly at concentrations as low as 30 *μ*M. Concentrations in the 40–80 *μ*M range significantly reduced cell viability; however, cell toxicity of TMP significantly increased. Then, we chose 30 *μ*M TMP to intervene in the cells in further experiments. A TMP time-response curve was created, and 12 h of TMP was found to be the optimum time, which was used in all of the experiments. As shown in Figure 1, western blot analysis for Smo, Patched, and Hhip protein levels showed inhibition of Hh signaling pathway with a dose-response pattern.

### 3.2. TMP Decreases Cyclin D1, Cyclin E1, and CDK2 Activities in HSC-T6 Cells

**Figure 2:**
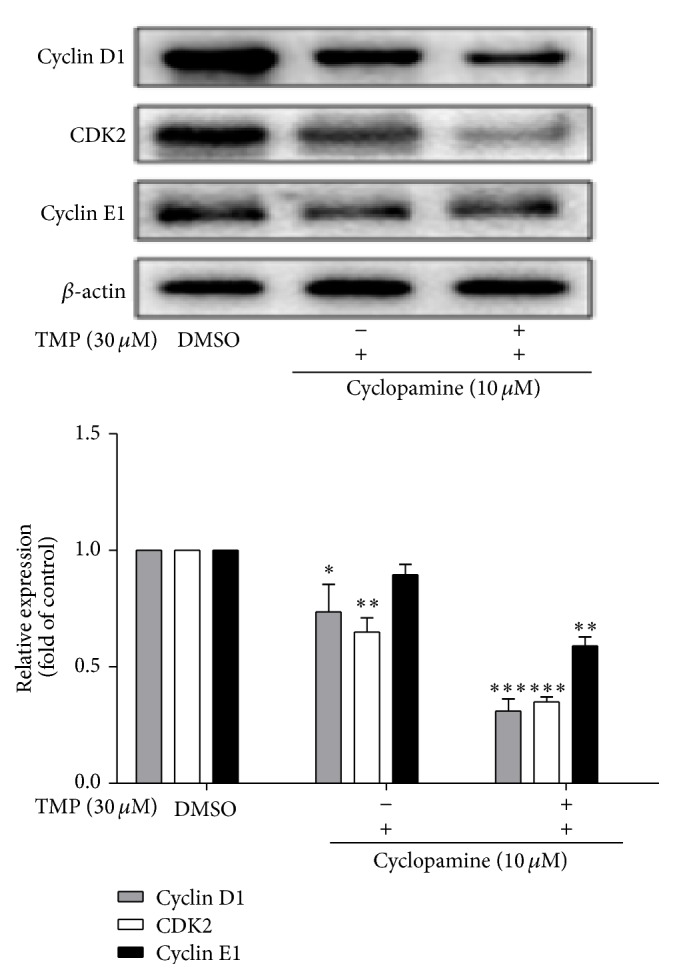
Effects of TMP on the activities of Cyclin D1, Cyclin E1, and CDK2 in HSC-T6 cells. Data were reported as means ± SD. For statistical analysis, ^*∗*^ *p* < 0.05, ^*∗∗*^ *p* < 0.01, and ^*∗∗*^ *p* < 0.001 represent significant difference.

Liver fibrosis is associated with the proliferation of HSCs, and the cell cycle of activated HSCs is abnormal. Cyclin D1, Cyclin E1, and CDK2 play essential roles in cell proliferation. Thus, the question is whether Cyclin D1, Cyclin E1, and CDK2 activities are inhibited by TMP treatment. Western blot analysis showed that TMP (30 *μ*M) and cyclopamine (10 *μ*M, specific inhibitor for Hh signaling pathways) significantly reduced the Cyclin D1, Cyclin E1, and CDK2 protein expression (Figure 2). The results showed that TMP significantly induced cell cycle arrest by blocking Hh signaling pathways.

### 3.3. TMP Induces HSC-T6 Cell Apoptosis

**Figure 3:**
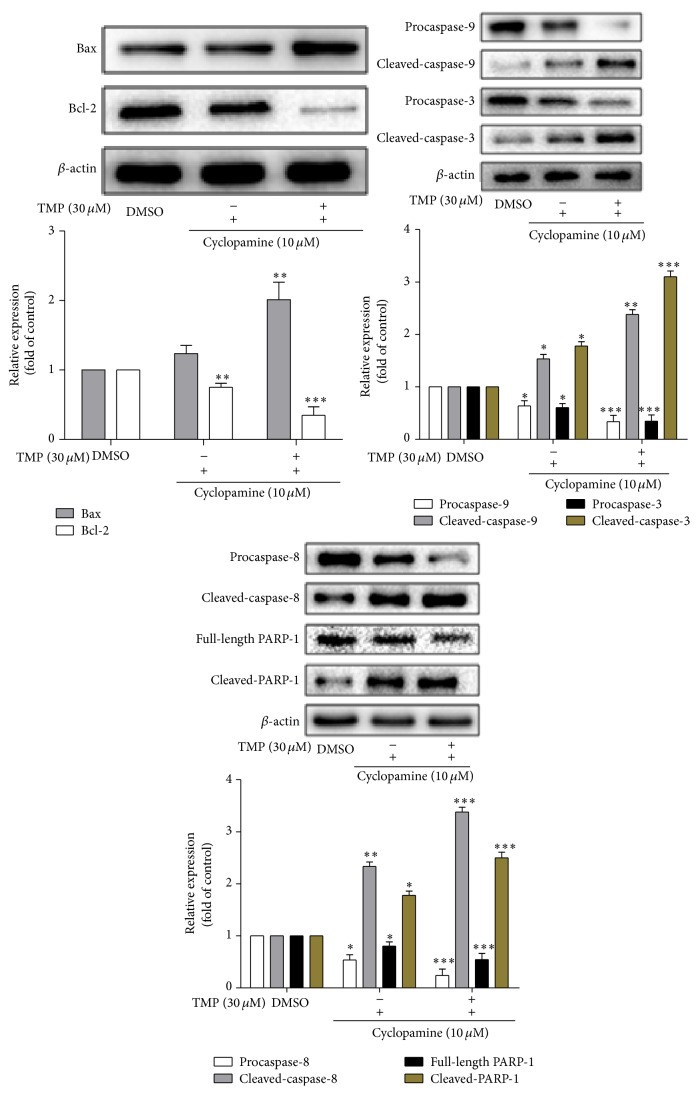
The effects of TMP on caspase activation and on the levels of Bcl-2/Bax proteins expressed in HSC-T6 cells. Data were reported as means ± SD. For statistical analysis, ^*∗*^ *p* < 0.05, ^*∗∗*^ *p* < 0.01, and ^*∗∗*^ *p* < 0.001 represent significant difference.

Caspases have been recognized as important mediators of apoptosis through the cleavage of various cellular substrates. Thus, we assess the effects of TMP on caspase activation by western blotting analyses. The results are as shown in Figure 3; TMP and cyclopamine treatment induced HSC-T6 cell apoptosis by activating caspase-3, PARP-1, caspase-8, and caspase-9. Furthermore, TMP and cyclopamine have synergistic effects on the apoptosis of HSC-T6 cells.

Bcl-2 and Bax, members of the Bcl-2 family of proteins, are antiapoptotic and proapoptotic factors, respectively. Therefore, we investigated whether the ratio of Bcl-2/Bax is inhibited by the pretreatment of cells with TMP. The results showed that TMP has also been shown to decrease the expression of Bcl-2 and increase the expression of Bax in HSC-T6 cells.

### 3.4. TMP Alters the Expression of Some Fibrotic Marker Proteins in HSC Activation

**Figure 4:**
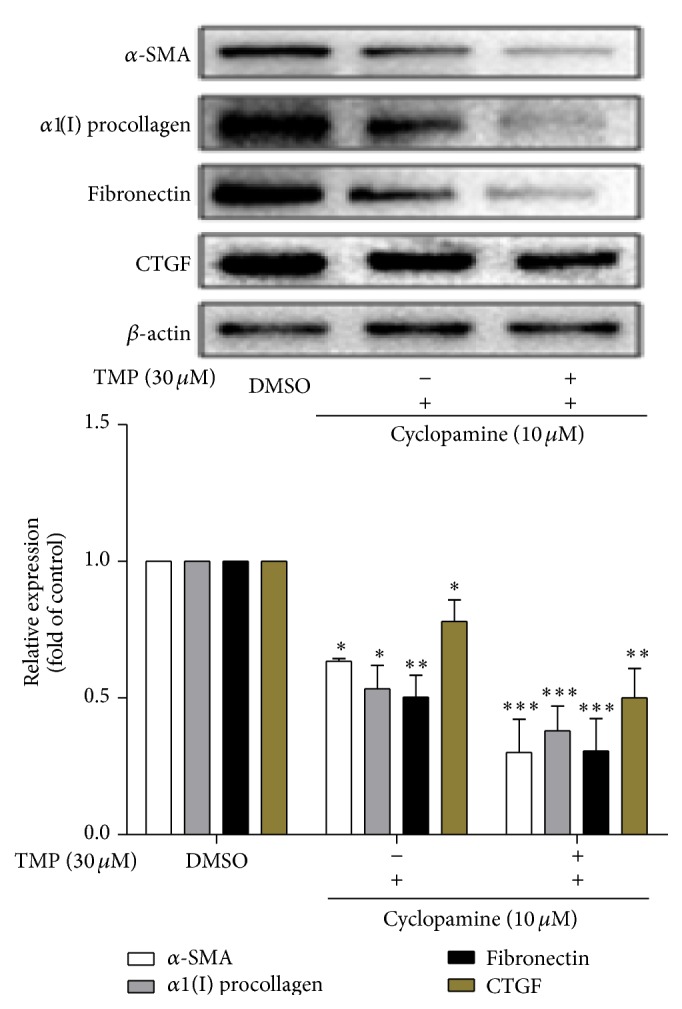
The effects of TMP on the levels of *α*-SMA, *α*1(I) collagen, fibronectin, and CTGF proteins expressed in HSC-T6 cells. Data were reported as means ± SD. For statistical analysis, ^*∗*^ *p* < 0.05, ^*∗∗*^ *p* < 0.01, and ^*∗∗*^ *p* < 0.001 represent significant difference.

*α*-SMA is the primary marker of HSC activation, whereas *α*1(I) collagen and fibronectin are the major components of ECM secreted by HSC. Western blot assay results indicated that TMP can inhibit the expression of *α*-SMA, *α*1(I) collagen, and fibronectin. The expression of CTGF, which mediates development and secretion of ECM and plays a key role in HF development, was found to increase HSC activation (Figure 4). In summary, this study found that TMP can inhibit the expression of CTGF, and the inhibitory effect was intensified after the application of joint treatment with TMP and cyclopamine.

## 4. Discussion

The Hh signal response primarily relies on the control of membrane proteins Ptc and Smo [13, 14]. The study showed that the expression of both proteins can be significantly inhibited by TMP in a dose-dependent manner. Therefore, the Hh signaling pathway may be the key hub to inhibit HSC activation. In the experiment, TMP showed the potential to inhibit the activities of Cyclin D1, Cyclin E1, and Cyclin-dependent kinase CDK2 and to change the HSC cycle by inhibiting the proliferation of HSC. Cell cycle arrest was also associated with HSC apoptosis, which is very important to maintain the integrity of the organism and selectively remove excessive or damaged cells. In the report, the treatment of HSC by TMP showed that the ratio of Bcl-2/Bax was downregulated, whereas caspase family proteins were activated. These results indicated that HSC apoptosis can be induced by TMP. Furthermore, TMP can reduce the secretion of *α*1(I) procollagen, fibronectin, and CTGF in ECM. The TMP can then stop and reverse the development of HF by inhibiting the HSC activity. Synergistic effects were presented after TMP combined with Hh signaling pathway, and inhibitor cyclopamine was applied.

TMP can improve hepatic fibrosis; however, TMP may exert an antihepatic fibrosis effect by blocking the Hh signaling pathway at G0/G1 phase in cell cycle arrest and triggering caspase-dependent HSC apoptosis. Recent studies reported that downstream target genes of the Hh signaling pathway were closely related to those of other signaling pathways, such as Wnt and TGF-*β*, which may be involved in the regulation of these signaling pathways [15]. Thus, investigation of TMP in regulating the Hh signaling pathway may establish a new approach for hepatic fibrosis treatment.
